# The genetics of falling susceptibility and identification of causal risk factors

**DOI:** 10.1038/s41598-023-44566-w

**Published:** 2023-11-09

**Authors:** Matt C. Smith, Jessica O’Loughlin, Vasileios Karageorgiou, Francesco Casanova, Genevieve K. R. Williams, Malcolm Hilton, Jessica Tyrrell

**Affiliations:** 1 Genetics of Complex Traits, College of Biomedical and Clinical Sciences, Faculty of Health and Life Sciences, University of Exeter, Exeter, UK; 2 Public Health and Sports Sciences Department, University of Exeter Medical School, Exeter, UK; 3 Clinical and Biomedical Science, University of Exeter Medical School, Exeter, UK

**Keywords:** Genetics, Population genetics, Epidemiology

## Abstract

Falls represent a huge health and economic burden. Whilst many factors are associated with fall risk (e.g. obesity and physical inactivity) there is limited evidence for the causal role of these risk factors. Here, we used hospital and general practitioner records in UK Biobank, deriving a balance specific fall phenotype in 20,789 cases and 180,658 controls, performed a Genome Wide Association Study (GWAS) and used Mendelian Randomisation (MR) to test causal pathways. GWAS indicated a small but significant SNP-based heritability (4.4%), identifying one variant (rs429358) in *APOE* at genome-wide significance (P < 5e-8). MR provided evidence for a causal role of higher BMI on higher fall risk even in the absence of adverse metabolic consequences. Depression and neuroticism predicted higher risk of falling, whilst higher hand grip strength and physical activity were protective. Our findings suggest promoting lower BMI, higher physical activity as well as psychological health is likely to reduce falls.

Humans utilise visual, proprioceptive and vestibular systems to stand upright and maintain balance^1,2^. These systems are critical for human function, with loss of balance and disorders of the balance system (e.g. vestibular disorders) increasing an individual’s risk of falls. Falls and fall related fractures represent a huge health and economic burden, with strong associations with increased morbidity, mortality and disability^3,4^. Determining factors that predict falls is challenging as they can be both intrinsic and extrinsic^5^ and the available evidence in this field remains limited, with the majority of studies focusing on older individuals.

Previous observational studies and several systematic reviews^3,6–10^ have highlighted the importance of a range of traits including adiposity, physical activity, previous falls, gait problems, vertigo, visual impairment, fear of falling, cognitive impairments and mental health problems in predicting falls. However, the majority of studies have focused on older individuals (60 years +) and not used methods that enable causal inference. Therefore, the results are not generalisable to the wider population and may be subject to confounding and reverse causality^11,12^. Understanding factors that cause falls is important, particularly when considering the possibility of modifiable risk factors that could potentially be targeted for prevention or intervention strategies.

A previous study by Trajanoska et al.^13^, performed a genome wide association study (GWAS) of self-reported falls using data from the UK Biobank. They demonstrated that despite the heterogeneity of factors contributing to fall risk, genetics was found to play a role, accounting for 2.7% of the overall susceptibility to falls. This study also showed positive genetic correlations between self-reported falls and fractures, insomnia, neuroticism, depressive symptoms and body mass index (BMI), as well as inverse correlations with muscle strength and intelligence. The falls measure used was a very broad, self-reported metric and as a result captured a wide variety of falls including falls related to accidents and falls not specific to balance issues.

Ideally, evidence of causal effects comes from well conducted randomised control trials (RCTs). However large-scale RCTs cannot always be performed because they can be costly, impractical, or even unethical^14^. One of the alternatives is to perform MR analyses that are similar to RCTs in terms of study design. MR (Fig. 1) uses genetic variation as a natural experiment to investigate the causal relations between potentially modifiable risk factors and health outcomes in observational data^14^.

**Figure 1:**
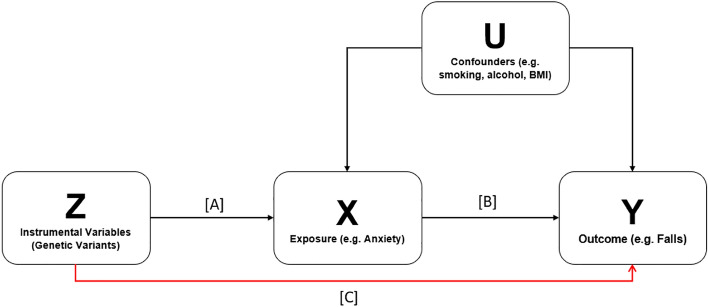
Directed acyclic graph (DAG) showing the relationship between the instrumental variables (IV), exposure, confounders and outcome. The IV must not have a direct effect on the outcome or the confounders for the assumptions of MR to be withheld. The IV may only act through the exposure. (**A**) describes the association between the exposure (X) of interest and genetic variants (Z) in the genome. (**B**) describes the observational.

A previous MR study provided evidence that higher BMI causes higher risk of falling and stronger hand grip reduces fall risk^13^. However, this study used a broad, self-reported, metric for falls and there are still many unanswered questions regarding the potential factors that have been suggested to contribute to the occurrence of falls^13^. For example, there is conflicting observational evidence for a role of mental health including depression, anxiety and neuroticism in fall risk^15,16^. There is observational evidence suggesting adverse mental health raises fall risk. However, clinical interventions aimed at reducing falls via mental health treatment has not proved successful^16,17^.

In this study, we aimed to utilise hospital and primary care records from the UK Biobank to define a binary balance related falls outcome. With this metric, our objective is to conduct a GWAS to explore genetic correlations. Additionally, we will employ multiple MR methods to assess potential causal relationships between a variety of predictors. These predictors have been selected based on prior literature and will be examined in relation to our falls metric.

## Methods

### The UK Biobank

The UK Biobank is a health resource with extensive phenotypic and genetic data available for over 500,000 individuals, who were aged between 37 and 73 at recruitment (from 2006 to 2010). Full details of this study are available^18,19^. Genome-wide genetic data was available for all participants, with approximately 850,000 variants directly measured using the UK Biobank Axiom (~ 450,000 individuals) or the UK BiLEVE (~ 50,000 individuals) array. These were then imputed using the UK10K and 1000 genome reference panels. Extensive quality control was carried out by UK Biobank, as previously described^19^. We defined ancestry using principal components in the 1000 Genomes Cohort as previously described^20^. We included up to 451,036 UK Biobank participants genetically similar to the 1000 Genome European superancestry group which included 209,298 participants with data linked to GP codes. Genetic similarity was derived using principal component analysis using the 1000 Genomes Cohort as our reference, as previously described^20^.

### Exposure and outcome measures

#### Exposures

Potential exposures were considered if there was a) prior evidence in the scientific literature of an association with falling and b) known genetic variants associated with the exposure for use in MR (see Genetic Analysis section). Briefly, the exposures fit into three categories: body mass (BMI and adiposity), physical activity (hand grip max strength, measured physical activity and sedentary time) and mental health (depression, anxiety and neuroticism). A full list of the exposures considered in this study is available in Table 1. For adiposity, we included genetically defined favourable and unfavourable adiposity (FA and UFA respectively). FA variants are associated with higher BMI and body fat, but lower risk of heart disease, diabetes and dyslipidaemia. In contrast UFA, is associated with higher fat and poorer metabolic profile^21^. These variants will allow us to test if it solely something about being fatter that increases falls risk or whether this is driven by poorer metabolic health^22^.

**Table 1:** List of considered exposures with previous evidence of association and known genetic variants.

Exposures	Previous evidence of falls association	Reference for observational associations	Primary MR Method used and justification	Other MR methods used	Primary GWAS reference
Mental health					
Depression	Yes, evidence of positive correlation	Alenazi et al.^8^	MRlap—largest sample size from summary statistics, both include UK Biobank	1SMR, 2SMR	Howard et al.^64^
Neuroticism	Yes, evidence of positive correlation but association may be mediated by depression and fear of falling	Turunen et al.^56^	MRlap—largest sample size from summary statistics, both include UK Biobank		Luciano et al.^72^
Anxiety	Yes, evidence of positive correlation	Hallford et al.^57^	MRlap—largest sample size from summary statistics, both include UK Biobank		Otowa et al.^65^
Social/behavioural					
Smoking status	Yes, evidence of a positive correlation but observational literature is inconsistent	Ek et al.^71^	1SMR—requires stratification by smoker status		Ware et al.^73^
Alcohol consumption	Yes, evidence of positive correlation	Tan et al.^70^	1SMR—requires stratification by smoker status	2SMR	Clarke et al.^74^
Education	Yes, evidence of a higher risk of falls in those with low educational attainment compared to college educated	Kim et al.^76^	MRlap—largest sample size from summary statistics, both include UK Biobank	2SMR	Marioni et al.^75^
Physical activity	Yes, evidence has shown a protective effect of physical activity on the risk of falls	Heesch et al.^77^	MRlap—largest sample size from summary statistics, both include UK Biobank		NA (Internal)
Sedentary time	Yes, evidence of positive correlation, however a meta-analysis shows moderate heterogeneity (36%)	Thibaud et al.^69^	MRlap—largest sample size from summary statistics, both include UK Biobank		NA (Internal)
Physical characteristics					
BMI	Yes, evidences shows a non-linear association with very low and very high BMI being associated with a higher risk of falls	Aune et al.^62^	2SMR—good instrument available that excludes UK Biobank	1SMR	Locke et al.^67^
Hand grip max strength	Yes, evidence of a protective effect of hand grip strength on the risk of falls	McGrath et al.^68^	MRlap—largest sample size from summary statistics, both include UK Biobank	1SMR	Willems et al.^66^
Favourable adiposity	Not published		2SMR—requires use of pre-defined SNP set		Martin et al.^50^
Unfavourable adiposity	Not published		2SMR—requires use of pre-defined SNP set		Martin et al.^50^

#### Outcomes

We derived a binary fall outcome metric using the hospital episode statistics (HES) and general practitioner (GP) records available in the UK Biobank^23^. First, we extracted 137 ICD-10 codes and 84 GP read2/read3 codes (Supplementary Table 1). We then reviewed these codes with a Consultant Otolaryngologist Hilton M) and removed fall codes that were unlikely to be related to balance issues (e.g. codes relating to diving or falling into water; Supplementary Table 1). The HES data included falls reported between 1995 and 2021 (mean age of falling = 69.8 years) and the GP data included falls reported between 1955 and 2017 (mean age of falling 61.2 years). For completeness, we also tallied the number of falls for each individual that had at least one fall code in their records. Any occurrences that occurred on the same date but were reported using a different code were counted as one fall rather than multiple falls.

We defined controls as individuals with a HES or GP record and no recorded code relating to falls (including all fall codes; Supplementary Table 1). Our main analyses focused on the combined falls metric derived using both the HES and GP records, with 20,789 individuals reporting a balance related fall and 180,658 controls. We also repeated our analyses using a GP only derived metric (11,654 falls and 197,644 controls) and our HES only derived metric (23,236 falls and 408,650 controls) to test if the HES and GP records capture different types of falls. Finally, we derived a count of falls in the 20,789 reporting at least one fall.

During the baseline interview, all participants of the UK Biobank were asked to self-report falls within the last year. Although we did not use this metric to derive our balance related falls phenotype, we did check the overlap of cases with our metrics. Among our cases, 6447 individuals (31%) also reported a fall in the self-report data, while only 195 individuals (0.001%) among our controls reported a self-reported fall.

### Statistical analyses

#### Observational associations

To test the association of a range of exposures with our binary falls metric we used logistic regression adjusting for age at UK Biobank baseline assessment and sex. To test if factors that predicted our binary falls metric also predicted number of falls, we used a Poisson model.

#### Genome-wide association analysis

Association analyses used imputed genotypes available from the UK Biobank^24^. Variants were excluded if imputation quality (INFO) was < 0.3 or the minor allele frequency (MAF) was < 0.1%. All individual variant association testing was performed using REGENIE (Version 2)^25^. This method accounts for population structure and employs a Firth logistic regression to account for unbalanced case–control phenotypes, such as our falls phenotype. A number of covariates were included at runtime: age, sex, UK Biobank assessment centre and genotyping platform (categorical UKBiLEVE array, UKB Axiom array interim release and UKB Axiom array full release).

#### Genetic correlations

We used linkage disequilibrium (LD) score regression^26^ to quantify the genetic overlap between a) our fall phenotype and b) 12 traits of interest with 3 publicly available GWASs and 7 internally run GWASs. This method uses the GWAS summary statistics from two GWASs and regresses them against a measure of how much variation each single nucleotide polymorphism SNP tags (its LD score). Correlations were reported if they reached a Bonferroni corrected *P* value (number of tests = 12; *p* < 0.0042).

#### Mendelian randomisation (MR*)*

We undertook MR analyses to further test the causal relationships between the 13 exposure traits (decided a priori on the grounds that they are common exposures with observational evidence of an association). Where possible we also tested the bidirectional association, with falls as the exposure. We performed several different types of MR analyses to account for data availability and various statistical biases in our models. Results are presented from the most appropriate method for each exposure (Table 1) but generally results were consistent across the different methodologies. The different MR methods are summarised below:

##### One-sample MR

We performed one sample MR analyses at the individual level within up to 430,944 unrelated UKB participants. The effects of grip strength, alcohol consumption, BMI and depression (defined using the Composite International Diagnostic Interview (CIDI) and the Patient Health Questionnaire (PHQ-9)) on falls were estimated with the two-stage least squares^11^ approach and observational associations were reported for reference. In these analyses we also considered our fall count metric as an outcome.

In all analyses, the exposure phenotypes were regressed on the genetic instruments and the predicted values were then used in the second stage to obtain the unconfounded estimate of the exposures’ causal effects. Where possible, no or minimal overlap of the gene selection study with UKB was preferred. However, this was not always possible (e.g. hand grip exposure also included UKB), in these cases MR Lap (see below) was also performed to account for sample overlap. We considered three options for the second stage of MR, based on the nature of the distribution of the falls phenotype. For our primary binary outcome logistic models were used. For the falls count metric, we utilised either a linear model or a Poisson model. It was anticipated that the latter approach would be the most appropriate as the falls count metric generally follows a Poisson distribution.

##### Two sample MR

The two-sample MR analyses used summary-level data from the REGENIE GWAS of the falls traits. Known SNPs for each exposure were extracted from the falls GWAS results, representing the association of the outcome and exposure-trait-SNP, whilst published coefficients from the primary GWAS represent the exposure association with the exposure-trait-SNP. Using a custom pipeline, we performed four two-sample MR methods: inverse-variance weighting (IVW); MR-Egger^27^; weighted median (WM); penalised weighted median (PWM)^28^. For the two-sample analyses the IVW approach represents our main analyses, with MR-Egger, WM and PWM used as sensitivity analyses to account for unidentified pleiotropy that could bias our results^29^.

##### MR lap

Several of our exposures included UK Biobank in the primary GWAS (Table 6) This can induce bias to MR analyses and contribute to Winner’s curse. To account for this, we utilised MRLap (https://github.com/n-mounier/MRlap)^30^. MRLap is designed to correct for weak instrument bias and winner’s curse, whilst also accounting for sample overlap. MRLap provides observed (uncorrected) and corrected IVW results and quantifies the difference between the observed and corrected effects. Detailed information about the method and its approach to adjusting for biases can be found in Mounier et al. Analyses performed with MRLap are summarised in Table 6. In addition, we tested the bidirectional relationship between falls and our exposures using this method (F Statistic = 21.7).

In both the two-sample MR and MR Lap where we had a binary exposure with summary statistics based on a log odds ratio (ln(OR)), our effect estimates represent a change in fall risk per change in the binary exposure on a log odds scale. Therefore for interpretation we calculated the odds of falling per doubling in the genetic risk for the binary predictor by multiplying the causal estimate by 0.693 (= ln2)^31^.

### Sensitivity analysis in the MR framework

#### Sex-stratified MR to eliminate pleiotropy

In the one sample setting we also employed a novel approach to eliminate pleiotropy, which exploits sex specific associations^32^The genetic associations for grip strength are known to differ substantially between men and women based on previous evidence^33^ The instrument selection involved identifying SNPs that are stronger for one sex (gene-sex interaction), subtracting them, and extracting these from sex-specific falls GWAS.

#### Alcohol and smoking analyses

Alcohol and smoking univariable analyses were also performed in several subgroups of individuals, stratified by either alcohol drinking status (never versus ever) and smoking status (never, former and current). In never drinkers and never smokers we would not expect to see any association of our alcohol or smoking genetic instrument respectively.

All analyses were performed in R version 4.1.2.

## Results

Demographics of the falls cases and controls can be found in Table 2. Briefly, individuals with a recorded fall were older, more likely to be female, have a higher BMI and lower maximum hand grip strength. Individuals reporting falls were also more likely to have a lower educational attainment and were more likely to report having depression and anxiety.

**Table 2:** Demographics of European Individuals in UK Biobank with and without fall codes.

Trait	Falls	Control	*p*-Value*
N	20,789	180,658	
Mean age at baseline in years (SD)	60.8 (7.14)	56.7 (7.99)	< 2e−16
Sex (male)	7900 (38%)	83,103 (46%)	< 2.2e−16
Mean BMI (SD)	28.3 (5.37)	27.4 (4.71)	< 2e−16
Mean waist to hip ratio (SD)	− 0.862(3.27)	− 1.53 (2.95)	< 2e−16
Mean international physical activity Questionnaire (IPAQ) score (SD)	25.9 (10.7)	30.3 (11.3)	< 2e−16
Mean hand grip max strength (SD)	5945 (28.6%)	43,719 (24.2%)	1.15E−11
Number reporting depressed ever (%)	3.29(3.03)	2.99 (2.97)	3.94E−16
Mean CIDI MDD severity score (SD)	3.49 (4.36)	2.78 (3.70)	< 2e−16
Mean PHQ9 severity score (SD)	2.57 (3.86)	2.15 (3.39)	< 2e−16
Mean GAD7 Severity Score (SD)	13.5 (5.36)	15.0 (5.10)	< 2e−16
Mean number of years of education (SD)	14.3 (20.9)	14.9 (20.2)	0.001
Mean units of alcohol consumed weekly (SD)	0.629 (0.687)	0.538 (0.659)	0.001
Current smoker status (SD)	8.29 (12.2)	6.35 (10.5)	< 2e−16
Mean cigarettes per day in smoker (SD)	7.33 (1.20)	7.42 (1.13)	6.86E−04
Mean townsend deprivation index (TDI) score (SD)	0.879 (0.0925)	0.870 (0.0898)	< 2e−16

*p-Values for all measures were calculated using a logistic regression model. Age at baseline and sex were included as covariates.

Of the 20,789 of individuals with a known fall, 6444 (31%) had more than one fall (range 2–59; Supplementary Fig. 1). Individuals reporting more falls were older, more likely to be female, have a higher BMI, a lower hand grip strength and spend less time doing physical activity. They were also more likely to have a lower educational attainment and more likely to report depression and anxiety (Table 3).

**Table 3:** Observational analysis representing the odds of falling per unit or SD change in the exposure metric against binary, fall count with and without controls.

Exposure	Binary	
Falls	OR (95% CI)	*p*-Value
Mean BMI*	1.169 (1.152–1.186)	< 2e−16
Mean waist to hip ratio*	1.297 (1.272–1.323)	< 2e−16
Hand grip strength*	0.687 (0.673–0.701)	< 2e−16
Times measuring > 100 mg physical activity*	0.795 (0.764–0.828)	< 2e−16
Times measuring > 40 mg physical activity*	0.824 (0.792–0.858)	< 2e−16
International physical activity questionnaire (IPAQ)*	0.938 (0.924–0.953)	1.12E−15
Depressed ever	1.382 (1.291–1.478)	< 2e−16
CIDI major depressive disorder severity (CIDI MDD)	1.050 (1.039–1.061)	< 2e−16
Public health questionaire severity (PHQ9)	1.062 (1.054–1.069)	< 2e−16
Generalised anxiety disorder (GAD7)	1.046 (1.038–1.054)	< 2e−16
GAD ever	1.678 (1.553–1.802)	3.76E−16
Number of years in education	0.972 (0.969–0.975)	< 2e−16
Units of alcohol consumption per week*	0.962 (0.937–0.989)	0.00513
Smoker status	1.245 (1.218–1.273)	< 2e−16
Cigarettes per day*	1.160 (1.141–1.179)	< 2e−16

*represents per change in standard deviation. Odds ratio represents an odds increase or decrease of a fall per unit or standard deviation change in exposure. All regressions using the binary falls metric as outcome uses a logistic model. All models were adjusted for age and sex.

### Observational evidence provided further evidence our exposures were associated with balance related falls

**Figure 2:**
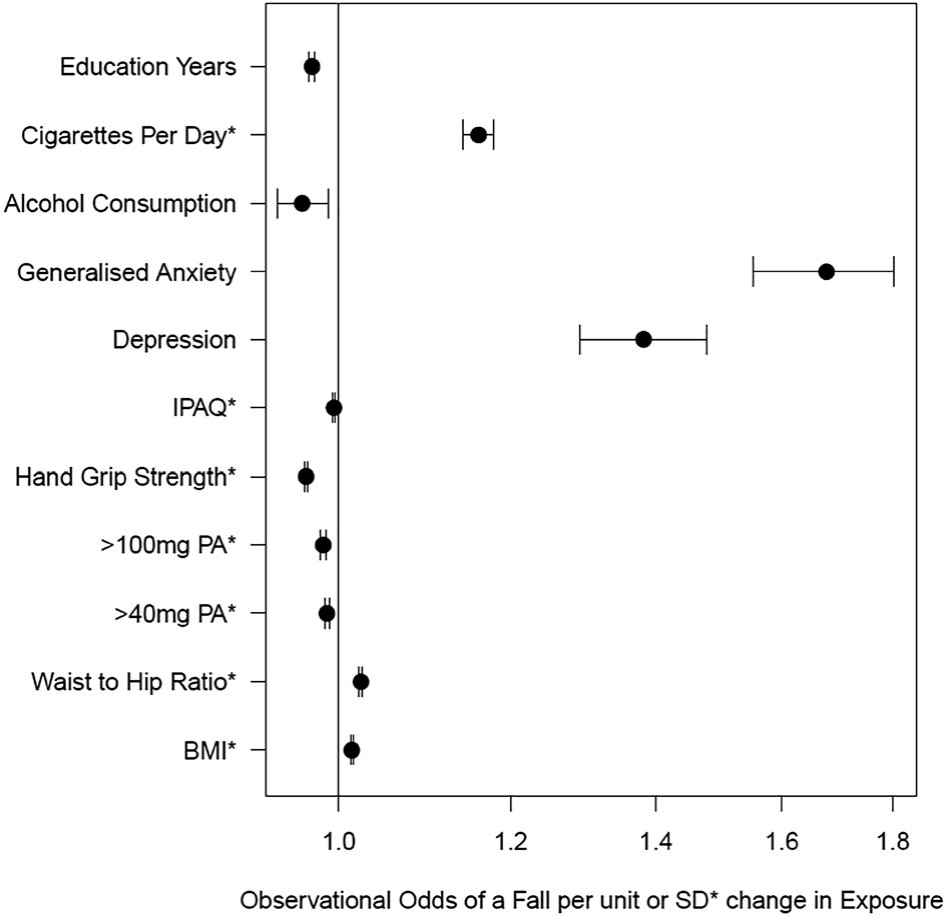
Forest plot representing the observational odds of falling for three main exposure groups: body mass, physical activity and mental health metrics. (IPAQ: International Physical Activity Questionnaire) (> 40 & > 100 mg PA: The sum of all actigraphy measures greater than or equal to 40 and 100 milli-gravity respectively).

Observationally, several exposures were robustly associated with having a fall in UKB (Fig. 2; Table 3). For example, a one standard deviation (SD) higher BMI (4.8 kg/m^2^) was associated with 1.17 higher odds of falling (95% CI: 1.15, 1.19). In contrast a 1 SD higher hand grip strength (11.34 kg) was associated with 0.69 lower odds of falling (95% CI: 0.67, 0.70).

There was also observational evidence for a role of mental health in fall risk. Both depression status and severity associated with higher risk of falling. For example, depression status was associated with 1.35 higher odds of falling (95%CI: 1.29,1.42) and a one unit higher severity of depression was associated with 1.06 higher odds of a fall (95%CI: 1.05,1.07). Anxiety was also associated with higher fall risk (OR:1.68, 95% CI: 1.55,1.80), as was higher anxiety severity (OR: 1.05, 95%CI: 1.04,1.05).

Observationally higher physical activity (measured using self-report and accelerometery data) was associated with a lower risk of falling. A 1-SD higher moderate to vigorous physical activity (MVPA) and + 40milligravitiy activity (40 mg) was associated with 0.78 (95% CI:0.76, 0.83) and 0.82 (95% CI:0.79, 0.86) lower odds of a fall respectively. Furthermore, a 1-SD higher score (1.14) on the self-reported international physical activity questionnaire (IPAQ) score was associated with 0.94 (95% CI: 0.92, 0.95) lower odds of a fall.

Observational results were generally similar for HES only and GP only derived falls (Supplementary Table 2).

## GWAS of the balance falls metric identified one loci in the *APOE* gene

The genetic heritability of combined falls was 4.4% (2.8% for HES falls and 3.8% for GP falls). One locus was identified at genome wide significance (GWS; *p* < 5e−08) in our main analyses (Table 4). This mapped to the *APOE* gene, where rs429358 is a missense mutation, with the C-allele of the *APOE-ε4* genotype being a major risk factor for Alzheimer’s disease. Here, the C-allele also predicts a higher likelihood of falling. This variant has been identified in many GWAS studies including plasma levels^34^, LDL-cholesterol^35^, superior frontal cortical thickness^36^, Alzheimer’s disease^37^ and longevity^38^.

**Table 4:** Lead SNP hits in falls GWAS.

Fall Phenotype	SNP	Chromosome	Location (Bp)	A1/A2	Frequency	Beta (SE)	*p* Value*	Known associated signal
Binary Combined	rs429358	19	45,411,941	C/T	0.85	− 0.085 (0.015)	4.15E−09	Missense Variant in APOE-ε4 C-allele increase Alzheimer’s
Binary HES	rs429358	19	45,411,941	C/T	0.85	− 0.119 (0.013)	9.21E−20	Missense Variant in APOE-ε4 C-allele increase Alzheimer’s
Binary HES	rs2908008	7	120,959,062	T/C	0.44	0.058 (0.010)	3.51E−09	Close to *WNT16*. Associated with heel bone mineral density in FinnGen GWAS

When stratifying our analyses by age the lead SNP, rs429358, was associated with higher odds of a fall per risk increasing allele higher in both those over 65 (OR: 1.15; 95% CI:1.09, 1.20) and those 65 or under (OR: 1.05; 95% CI: 1.02, 1.09).

When looking at HES and GP falls alone, the *APOE* locus reached GWS in the HES derived fall metric, but not in the GP derived metric (*p* = 0.013). An additional GWS locus was identified in the HES alone fall phenotype, with variant rs2908008 in Chr 7 (Table 4). This variant is near the *WNT16* gene which has previously been associated with heel bone mineral density^39^ and is the top locus in the most recent falls GWAS data from FINNGEN (https://r9.finngen.fi).

There was no evidence of an association between the two previously published GWAS hits from the self-reported falls GWAS^13^ (*p* > 0.08; Table 5).

**Table 5:** Genetic correlations of binary falls against variables of interest.

Variable 1	Variable 2	rg*	*p*-Value	Source
Combined falls	HES falls	**0.858**	**2.19E−27**	Internal GWAS
Combined falls	GP falls	**0.995**	**1.37E−51**	Internal GWAS
HES falls	GP falls	**0.748**	**6.75E−06**	Internal GWAS
Combined falls	Published binary falls	**0.701**	**7.42E−17**	Trajanoska et al.^13^
HES falls	Published binary falls	**0.694**	**2.87E−11**	Trajanoska et al.^13^
GP falls	Published binary falls	**0.691**	**1.64E−18**	Trajanoska et al.^13^
Physical characteristics				
BMI	Combined falls	**0.3511**	**1.17E−11**	Yengo et al.^63^
WHR	Combined falls	**0.3674**	**1.93E−11**	Internal GWAS
Hand grip max	Combined falls	− **0.205**	**3.17E−05**	Internal GWAS
Mental health				
Depression	Combined falls	**0.4144**	**1.41E−11**	Howard et al.^64^
Anxiety	Combined falls	0.5374	0.007	Otowa et al.^65^
Neuroticism	Combined falls	**0.3199**	**3.19E−09**	Internal GWAS
Social/behavioural				
Physical activity	Combined falls	− 0.166	0.012	Internal GWAS
Education years	Combined falls	− **0.288**	**8.42E−08**	Internal GWAS
Alzheimer’s	Combined falls	− 0.036	0.63	Internal GWAS
Townsend deprivation index	Combined falls	**0.4136**	**4.78E−08**	Internal GWAS
Alcohol consumption	Combined falls	0.1325	0.21	Internal GWAS
Cigarettes per day	Combined falls	**0.0092**	**3.11E−07**	Internal GWAS

Significant values are given in Bold.

*rg is the proportion of the variance that two traits share due to genetic variants. Results in bold passed Bonferonni correction.

### Genetic correlations

After Bonferroni correction (*p* < 4.17E-03) we observed strong positive genetic correlations between the combined falls and the HES falls (r_g_: 0.86, *p*: 2.19e−27) and GP falls (r_g_: 0.99, *p*: 1.37e−51) data (Table 5). Similarly, a positive correlation was observed between HES falls and GP falls (r_g_: 0.7481, *p*: 6.75e−06) (Table 5). Our balance fall metric was also genetically correlated with the previously published self-reported falls GWAS (Table 5; r_g_: 0.70, *p*: 7.42e−17)^13^.

After Bonferroni correction the falls metric was positively genetically correlated with BMI, WHR, depression, neuroticism, Townsend Deprivation Index and cigarettes per day (Table 5). In contrast, negative genetic correlations were noted with hand grip strength, bone mineral density and years in education (Table 5). There was no evidence for a genetic correlation between falls and a) Alzheimer’s disease and b) anxiety (Table 5). The genetic correlations were repeated using the HES and GP derived falls metrics and were consistent (Supplementary Table 3).

### Mendelian Randomisation (MR)

#### MR provided further evidence for a role of higher BMI in fall risk and evidence this is not solely due to poorer metabolic health

**Figure 3:**
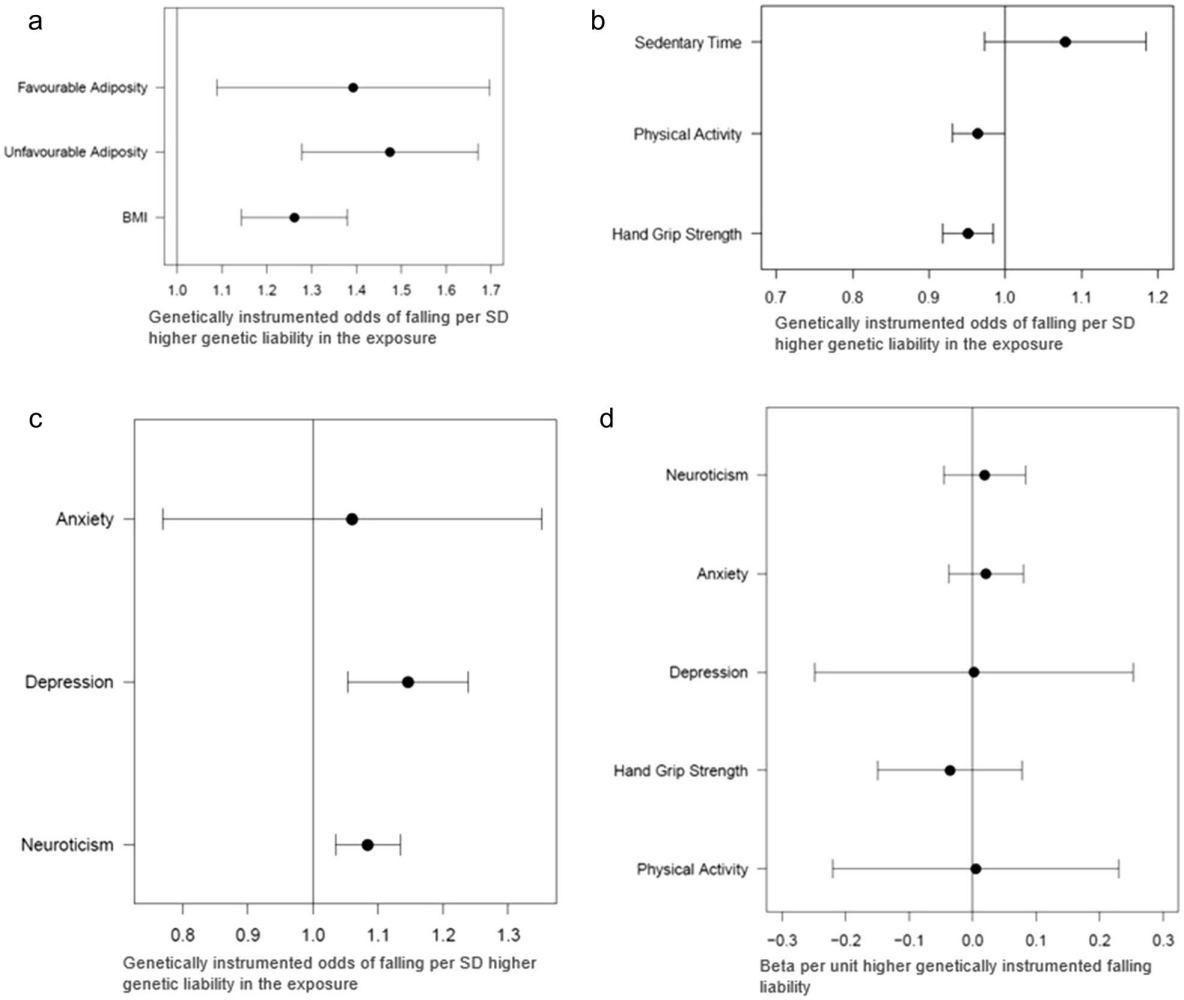
(**a**) Forest plot of 2-Sample MR results showing adiposity as the exposure and binary falls as the outcome. (**b**) Forest plot of MRLap results showing Physical Activity Phenotypes as the exposure and binary falls as the outcome. (**c**) Forest plot of MRLap results showing Mental Health Phenotypes as the exposure and binary falls as the outcome. (**d**) Forest plot of MRLap testing Bidirectional effect of Falls on metrics of interest. Binary Falls is treated as the exposure.

There was evidence from both one and two sample MR that higher BMI is causally associated with falling (Fig. 3a). For example, a 1-SD higher (4.5 kg) genetically instrumented BMI was associated with 1.26 higher odds of falling (95%CI: 1.12, 1.42). Whilst the more pleiotropy robust methods had wider uncertainty around the estimate, the associations were directionally consistent and there was no evidence of horizontal pleiotropy (*p* = 0.61; Table 6).

**Table 6:** Two sample MR and MR lap results for binary falls against phenotypes of interest.

Exposure	Outcome	Primary MR method	OR (95% CI)	*p* value
Physical characteristics				
BMI	Binary falls	2—Sample IVW	1.262 (1.145–1.379)	2.16E−04
Favourable adiposity	Binary falls	2—Sample IVW	1.393 (1.089–1.696)	0.029
Unfavourable adiposity	Binary falls	2—Sample IVW	1.475 (1.278–1.672)	4.00E−04
Hand grip strength max	Binary falls	MRlap	0.951 (0.919–0.983)	0.003

Mental health				
Depressed ever	Binary falls	2—Sample IVW	1.253 (1.049–1.458)	0.046
Neuroticism	Binary falls	MRlap	1.084 (1.035–1.134)	1.15E−08
Generalised anxiety	Binary falls	MRlap	1.060 (0.729–1.351)	0.70
PGC Major Depressive Disorder	Binary falls	MRlap	1.146 (1.045–1.256)	2.92E−05

Social/behavioural				
Physical activity	Binary falls	MRlap	0.965 (0.930–0.999)	0.038
Sedentary time	Binary falls	MRlap	1.078 (0.969–1.199)	0.17
Education years	Binary falls	MRlap	0.924 (0.890–0.959)	8.10E−06
Alcohol consumption	Binary falls	2-Sample IVW	1.449 (0.977–1.921)	0.18

Bidirectional associations				
Exposure	Outcome	Primary MR Method	Beta(SE) or OR (95%CI)	*p* Value
Binary falls	BMI	MRlap	0.965 (0.853–1.078)	0.89
Binary falls	Hand grip strength	MRlap	− 0.035 (0.058)	0.68
Binary falls	Physical activity	MRlap	0.005 (0.115)	0.95
Binary falls	Sedentary time	MRlap	− 0.063 (0.120)	0.45
Binary falls	PGC major depressive disorder	MRlap	1.002 (0.751–1.252)	0.99
Binary falls	Generalised anxiety	MRlap	1.021 (0.963–1.080)	0.31
Binary falls	Neuroticism	MRlap	0.019 (0.033)	0.56
Binary falls	Education years	MRlap	− 0.063 (0.036)	0.08

MRLap was used for all analyses where binary falls was the exposure as we needed a method that enabled weak instrument analyses.

Genetic variants that make an individual fatter but metabolically healthier (e.g. lower risk of type 2 diabetes, lower risk of cardiovascular disease), termed favourable adiposity, were associated with higher risk of falling (OR: 1.39; 95% CI: 1.09, 1.69). Similarly, there was evidence that genetic variants associated with higher unfavourable adiposity (i.e. higher body fat percentage, high risk of type 2 diabetes etc.) also predicted falling (OR: 1.48; 95% CI: 1.28, 1.67) (Table 6).

#### MR provided evidence that longer educational duration is protective from falls

Using MR Lap to account for the exposure and outcome data both coming from the UK Biobank we provide evidence that a longer educational duration is protective for fall risk. For example, a genetically instrumented one year longer educational duration associated with 0.93 lower odds of falling (95%CI: 0.90, 0.95). Results were similar using standard 2-sample MR and a smaller set of variants that excluded UK Biobank (Table 6).

#### Higher physical strength and activity protect from fall risk

There was evidence after accounting for sample overlap that higher hand grip strength was protective from falling. A 1-SD higher genetically instrumented hand grip (~ 11.3 kg) was associated with 0.95 lower odds of falling (95% CI: 0.92, 0.97; Table 6, Fig. 3b). There was no evidence of bias from overlapping samples (P = 0.27). A higher genetic liability to falling did not predict hand grip) (Table 6).

A higher genetically instrumented overall physical activity was nominally associated with lower risk of falling (OR: 0.96; 95%CI: 0.93, 1.00; *p* = 0.038). MRLap did not provide evidence that the overlapping samples or weak instrument biased the results. There was no evidence of a bidirectional association between PA and falls (*p* = 0.90; Table 6).

Higher genetically instrumented actigraphy measured sedentary time was not associated with fall risk (OR: 1.08 95%CI: 0.97, 1.20) and there was no evidence that a genetically higher fall risk predicted sedentary time (*p* = 0.45).

#### Major depression and neuroticism predict falling but no evidence for a role of generalised anxiety

MR-Lap provided evidence that major depression, with the most up to date Psychiatric Genomics Consortium (PGC) summary statistics^40^ predicted falling (Fig. 3c). A twofold higher genetic liability to major depression was associated with 1.15 higher odds of an individual falling (95% CI: 1.04, 1.26). There was some evidence that sample overlap biased our results and therefore the corrected effects are presented (corrected difference *p*-value = 0.003) (Table 6*.* There was no evidence for a bidirectional relationship between depression and falls (Table 6; Fig. 3d).

There was evidence that neuroticism was causally associated with falling. A one SD higher genetic neuroticism (~ 3.27 units) associated with 1.08 higher odds of falling (95% CI: 1.04, 1.12). The estimates obtained with MR Lap correction were similar to the uncorrected estimates. A higher genetic liability to falling did not associate with neuroticism. There was no evidence that a higher genetic liability to generalised anxiety predicting falling or vice versa (Table 6).

## Sensitivity analyses

### One-Sample MR

**Figure 4:**
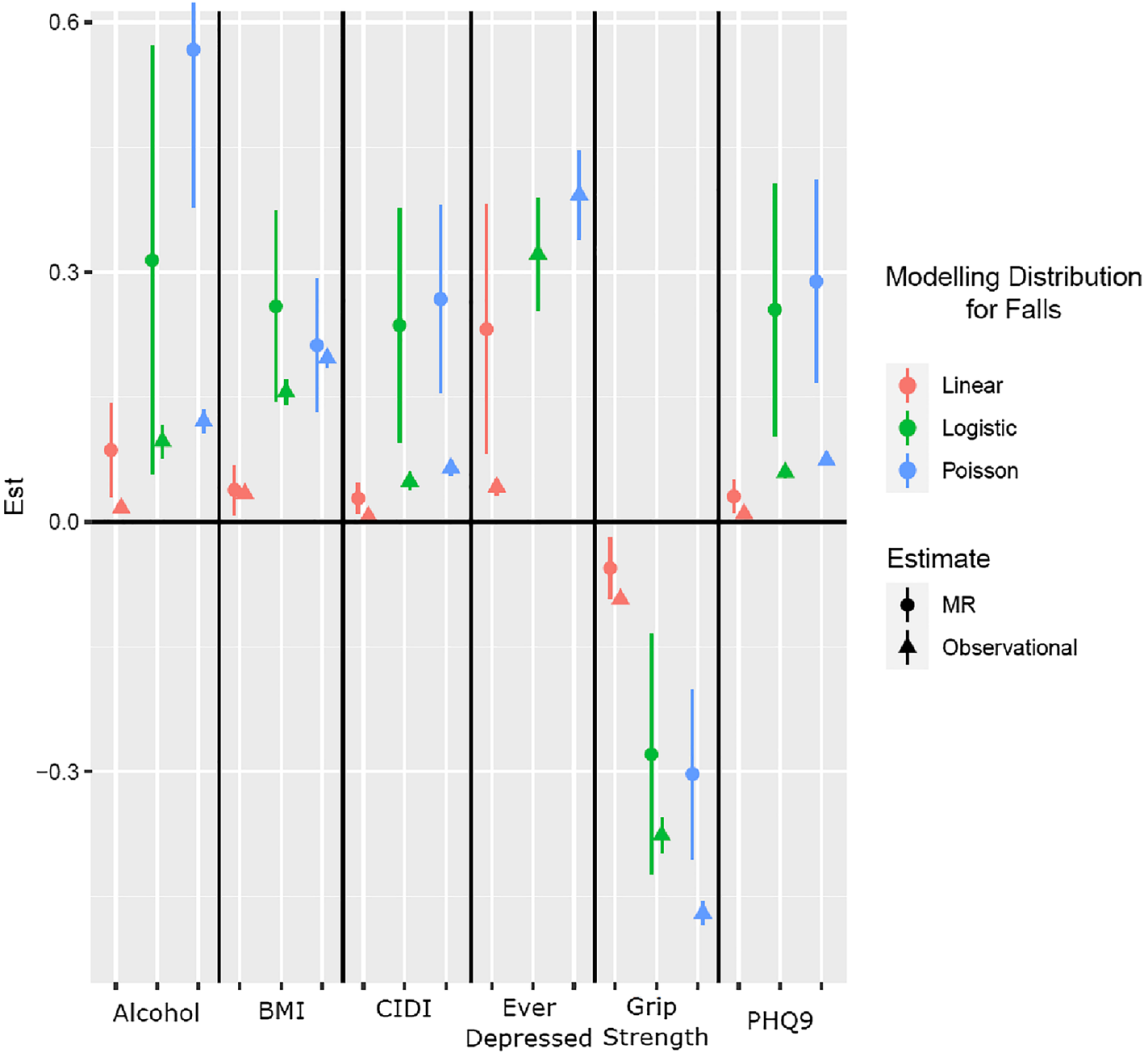
Forest Plot for the effects of alcohol, BMI, CIDI, CRP, ever depressed status, grip strength, and PHQ9 on falls. Univariable MR. Three different modelling choices for the outcome of falls are presented (linear for normal distribution, logistic regression for binomial distribution, and Poisson distribution), The respective observational associations are presented.

To further test causal pathways, we also performed some univariate one-sample MR using both the binary falls metric and the count of falls in our 20,789 falls cases (Supplementary Table 8). These models indicate that alcohol, BMI and all depression measures (major depression, major depression severity and current depression) are positively causally associated with falls, whereas grip strength seems to exert a protective effect (Fig. 4). The Poisson modelling approach provides point estimates that are numerically similar to the binomial distribution of the dichotomous falls phenotype, but all are more precise (confidence intervals for the Poisson model are on average 11.51% narrower than those of the logistic regression). Therefore, the second-stage Poisson distribution seems to improve power as it retains the information regarding the number of falls. In summary, alcohol, BMI, depression measures, and grip strength appeared to influence the binary falls metric and the count of falls, with effect sizes of similar magnitude to the ones reported above.

### Sex stratified MR

In the sex-stratified MR^32^, a total of 185 SNPs surpassed the F statistic threshold of 15 and were used as sex-specific instruments. There seems to be a protective causal effect of grip strength on risk of falls (RR: 0.74, 95% CI: 0.61, 0.91 *p*: 0.004).

### GP only and HES only falls MR

MR analyses were repeated using the GP and HES only derived fall phenotypes. Generally, the results were consistent (Supplementary Fig. 6). Increased sedentary time was associated with an increased risk of falls in the GP derived metric in contrast to the results of the combined and HES fall metrics. Physical Activity was found to be non-significant in the GP metric (OR:0.993; 95%CI:0.959,1.027) but significant in the combined (OR: 0.965; 95%CI: 0.930, 0.999) and HES (OR 0.973; 95%CI: 0.949, 0.997) metrics.

## Discussion

This study explored the genetic basis of a balance related fall phenotype derived from electronic health records. We provide further evidence that falls are a highly heterogenous polygenic trait with a small but significant SNP-based heritability (4.4%) and identified one locus in the *APOE* gene in GWAS. The study also used MR to test the causal role of a broad range of modifiable risk factors on falling, highlighting the importance of adiposity, depression and neuroticism in predicting falls, whilst improved physical functioning as defined by hand grip strength and physical activity protected from falls.

The GWAS signal identified in this study is a missense mutation in *APOE* gene (rs429358)^41^. The C-allele is the marker of the APOE-ε4 genotype which is a major risk factor for Alzheimer’s disease^42^, and here, was associated with higher odds of falling. This is a highly pleiotropic locus, which also associates with heart disease^43^, inflammation^44^ and dyslipidaemia^45^. Poorer health and frailty may explain the association with falling. A sensitivity analysis stratifying the association of the top hit by age (> 65 and ≤ 65) demonstrated that whilst the association was stronger in the older age group, an increased risk of falling remained in those under 65 (Supplementary Table 5).

The GWAS results for our balance related falls metric were correlated with the previous self-reported falls GWAS, however the variants previously identified by Trajanoska et al., were not associated with our falls metric (Table 5). These differences in findings may be explained by the different definitions of falling. Here, we used hospital and GP health records to code falls and restricted the falls codes to those that represent balance related falls. In contrast, the Trajanoska et al. used a self-reported fall metric, derived from positive responses to the question: “In the last year have you had any falls?” Methods of specifically identifying balance related falls, such as those used in the present study, are likely to capture more severe falls phenotypes, related to mechanisms of balance problems.

Genetic correlations provided evidence that risk of falling was positively correlated with BMI, WHR, depression and neuroticism, and negatively correlated with hand grip strength and bone mineral density. In these analyses there was no evidence of a genetic correlation between falling and physical activity, anxiety or Alzheimer’s disease. The correlation between falls, BMI hand grip strength is consistent with Trajanoska et al. However, our analysis also shows evidence of fall risk being genetically associated with lower bone mineral density in contrast to the findings of Trajanoska et al. which found no significant association.

Higher BMI was identified by MR as a risk factor for falling. This fits with evidence from several observational studies^46,47^ and the previous self-reported falls genetic study^13^. The relationship between BMI and falling may be explained by poorer health in those with a higher BMI and/or alterations to balance control^48,49^. More research is needed in this area as UK Biobank lacks power for analyses at lower BMIs. To gain further insight into the potential mechanisms by which higher BMI increases falls risk we used favourable and unfavourable adiposity genetic variants^50^, to instrument higher body fat percentage in the presence (unfavourable) and absence (favourable) of adverse metabolic consequences (i.e. type 2 diabetes, cardiovascular disease, dyslipidaemia). Both sets of genetic variants were associated with higher falls risk. This indicates that it is not solely the adverse metabolic health consequences of higher BMI driving the association, perhaps indicating the importance of BMI in balance control^51,52^.

Higher hand grip strength and physical activity were protective for falling. As previously noted by Trajanoska et al., lower hand grip was noted to increase the risk of falling. Here, we used novel MR methods that use sex stratified estimates to account for pleiotropy, providing further evidence for the protective role of hand grip on falls risk. Using MR approaches that account for weak instruments and sample overlap between the exposure and outcome summary statistics we provide evidence for a causal role in physical activity in reducing balance related falls risk which is consistent with previous work summarised in a systematic review of 8 studies investigating the use of physical activity programmes to increase balance performance and decrease fall risk^53^. This provides consistent evidence of the importance of muscle weakness as a risk factor for balance related falls and further highlights the benefits of remaining physically active throughout adult life.

We demonstrate that a higher genetic liability to depression increases an individual’s risk of falling. This fits with previous evidence^54^, including data from a meta-analysis of 17 prospective studies where depression was associated with falls^55^. There was no evidence that a higher genetic liability to falling predicted depression, but these analyses were limited by lower power. A higher genetic liability to neuroticism was also predictive of falls, adding to the observational evidence that neuroticism associates with falls^56^. Although it is still unclear as to whether the association between neuroticism and falls is mediated by depression as suggested by Turunen et al. or acts independently. Future work, should investigate the potential mediate effect of depression on neuroticism and falls, using multi-variable MR (MVMR) to determine if the association between neuroticism and falls is independent of depression.

Our results suggest that there is no evidence of a genetic correlation or a causal link between anxiety and risk of falling. This is in contrast to a recent meta-analysis of 18 studies which concluded that anxiety was positively associated with falls^57^. This may be partially explained by potential confounders such as fear of falling (FoF) and neuroticism driving the observed association between anxiety and falls. The literature surrounding FoF is sparce with measures of FoF such as the Falls Efficacy Scale International (FESI) lacking depth^58^ and studies generally focusing on older populations with specific conditions such as Parkinson’s^57^.

A recent qualitative study^59^ explored anxiety and FoF using semi-structured interviews. This study highlighted that the effect of FoF on fall risk is contextual and depends on an individual’s perception of control. Should one be worried about falling but this, in turn, increases their perception of potential risks and their own limitations then it acts protectively. However, should one have FoF, and this distracts the individual from their surroundings and potential risks then this is detrimental. This may explain the high heterogeneity (I^2^ = 71%) in the meta-analysis of the effects of anxiety on falls^57^ as well as the lack of causal evidence we see in this study. It should also be noted that rs429358, our lead hit, was associated with a lower odds of participation in the mental health questionnaires^60^ which may further bias the anxiety results towards the null.

This study has a number of strengths. It uses a refined balance falls metric in a large sample of individuals in the UK Biobank, rather than self-reported falls in the past year, which may be more reflective of health status over the last 12 months. However, we acknowledge that there are limitations using this derivation method. We were unable to capture falls that did not require a GP visit or hospital admission including visits to Accident and Emergency.

UK Biobank is not population representative, with a healthy volunteer bias^61^ and our analyses was restricted to those of European ancestry. Our analyses with actigraphy derived physical activity are susceptible to participation bias^60^. For example, individuals with actigraphy data had a fall prevalence of 7.7% compared to 10% in individuals without actigraphy data.

There are also inherent limitations of using binary outcome measures in MR analyses^31^. Genetic variants may influence the outcome through the associated continuous risk factor without changing the binary outcome itself^31^. However, we have observed that the genetic risk score for depression and other binary measures are associated with severity and is therefore representing an increased genetic liability to risk. We also acknowledge that GWASs of social traits such as depression and anxiety may be confounded by factors such as assortative mating and other indirect genetic effects.

This study has utilised several MR methods to account for potential biases associated with pleiotropy, sample overlap and weak instruments. In general, consistent results were seen across the different methods. Whilst each method has its own limitations, our use of multiple techniques, with consistent results, provides more confidence in our findings.

In conclusion, we have provided further evidence that fall risk is a heritable, heterogenous and polygenic trait, when using a balance related fall metric. We provide robust evidence for higher BMI (independent of metabolic consequences), depression, neuroticism and physical inactivity as risk factors for falling. Using multiple MR approaches, our study provides novel insights into factors that may cause falls, which can be used to optimise fall prevention strategies and to improve further research into balance control strategies.

### Supplementary Information

Supplementary Information.
