# Supplementary material for: The genetics of falling susceptibility and identification of causal risk factors
Source: Sci Rep. 2023 Nov 9;13:19493. doi: 10.1038/s41598-023-44566-w (PMC10636011; doi:10.1038/s41598-023-44566-w)
Supplement: Supplementary file 1 — Supplementary Information. [file 41598_2023_44566_MOESM1_ESM.docx]

| **Supplementary Table 1: ICD10 and GP fall codes used for fall phenotype definition** | | | | | |
| --- | --- | --- | --- | --- | --- |
| **All fall related GP codes** | **Included in Balance Phenotype** | **Reason for not including** | **All Fall related ICD10 codes** | **Included in Balance Phenotype** | **Reason for not including** |
| 16D.. | Yes |  | W00.. | No | Involve Ice and snow |
| 16D1. | Yes |  | W00.0 | No | Involve Ice and snow |
| 16D2. | Yes |  | W00.2 | No | Involve Ice and snow |
| 16D4 | No | No fear of falls | W00.9 | No | Involve Ice and snow |
| 15D5. | Yes |  | W01. | No |  |
| 16D6. | Yes |  | W01.0 | Yes |  |
| U10.. | Yes |  | W01.1 | Yes |  |
| U101. | Yes |  | W01.10 | Yes |  |
| U1010 | Yes |  | W01.11 | Yes |  |
| U1011 | No | None coded | W03. | No | Due to collision with other person |
| U1012 | No | None coded | W04. | No | Fall while being carried |
| U1013 | No | None coded | W05. | No | Invloves wheelchair |
| U1014 | Yes |  | W05.1 | No | Invloves wheelchair |
| U1015 | Yes |  | W05.2 | No | Invloves wheelchair |
| U1016 | Yes |  | W06. | No | Involves Bed |
| U1017 | No | None coded | W07. | No | Involves Chair |
| U101y | Yes |  | W08. | No | Involves other furniture |
| U101z | No | None coded | W09. | No | Involves playground equipment |
| U10A. | No | Falling on and from stairs | W09.0 | No | Involves playground equipment |
| U10A0 | No | Falling on and from stairs | W09.1 | No | Involves playground equipment |
| U10A1 | No | Falling on and from stairs | W09.2 | No | Involves playground equipment |
| U10A2 | No | Falling on and from stairs | W09.8 | No | Involves playground equipment |
| U10A3 | No | Falling on and from stairs | W10. | No | On and from stairs |
| U10A4 | No | Falling on and from stairs | W10.0 | No | On and from stairs |
| U10A5 | No | Falling on and from stairs | W10.1 | No | On and from stairs |
| U10A6 | No | Falling on and from stairs | W10.2 | No | On and from stairs |
| U10A7 | No | Falling on and from stairs | W10.8 | No | On and from stairs |
| U10Ay | No | Falling on and from stairs | W10.9 | No | On and from stairs |
| U10Az | No | Falling on and from stairs | W11. | No | Involves Ladder |
| U10J. | No | Falling on and from stairs | W12. | No | Involves Scaffolding |
| 16D5. | Yes |  | W13. | No | Out of or through building or structure |
| U10z. | Yes |  | W13.0 | No | Out of or through building or structure |
| U10z0 | Yes |  | W13.1 | No | Out of or through building or structure |
| U10z1 | No | non-specific fall | W13.2 | No | Out of or through building or structure |
| U10z2 | No | non-specific fall | W13.3 | No | Out of or through building or structure |
| U10z3 | No | non-specific fall | W13.4 | No | Out of or through building or structure |
| U10z4 | No | non-specific fall | W13.8 | No | Out of or through building or structure |
| U10z5 | No | non-specific fall | W13.9 | No | Out of or through building or structure |
| U10z6 | No | non-specific fall | W14. | No | From tree |
| U10z7 | No | non-specific fall | W15. | No | From Cliff |
| U10zy | No | non-specific fall | W16. | No | Diving or jumping into water |
| U10zz | No | non-specific fall | W16.0 | No | Diving or jumping into water |
| U10J. | No | non-specific fall | W16.01 | No | Diving or jumping into water |
| U10JO | No | non-specific fall | W16.02 | No | Diving or jumping into water |
| U10J1 | No | non-specific fall | W16.03 | No | Diving or jumping into water |
| U10J2 | No | non-specific fall | W16.1 | No | Diving or jumping into water |
| U10J3 | No | non-specific fall | W16.11 | No | Diving or jumping into water |
| U10J4 | No | non-specific fall | W16.12 | No | Diving or jumping into water |
| U10J5 | No | non-specific fall | W16.13 | No | Diving or jumping into water |
| U10J6 | No | non-specific fall | W16.2 | No | Diving or jumping into water |
| U10J7 | No | non-specific fall | W16.21 | No | Diving or jumping into water |
| U10Jy | No | non-specific fall | W16.22 | No | Diving or jumping into water |
| U10Jz | No | non-specific fall | W16.3 | No | Diving or jumping into water |
| TC… | No | None coded | W16.31 | No | Diving or jumping into water |
| TC0.. | No | Not on same level | W16.32 | No | Diving or jumping into water |
| TC00. | No | Not on same level | W16.33 | No | Diving or jumping into water |
| TC000 | No | Not on same level | W16.4 | No | Diving or jumping into water |
| TC001 | No | Not on same level | W16.41 | No | Diving or jumping into water |
| TC00z | No | Not on same level | W16.42 | No | Diving or jumping into water |
| TC01. | No | Not on same level | W16.5. | No | Diving or jumping into water |
| TC010 | No | Not on same level | W16. | No | Diving or jumping into water |
| TC011 | No | Not on same level | W16.0 | No | Diving or jumping into water |
| TC01z | No | Not on same level | W16.01 | No | Diving or jumping into water |
| TC02. | No | Not on same level | W16.02 | No | Diving or jumping into water |
| TC020 | No | Not on same level | W16.03 | No | Diving or jumping into water |
| TC021 | No | Not on same level | W16.1 | No | Diving or jumping into water |
| TC02z | No | Not on same level | W16.11 | No | Diving or jumping into water |
| TC0z. | No | Not on same level | W16.12 | No | Diving or jumping into water |
| TC5.. | Yes |  | W16.13 | No | Diving or jumping into water |
| TC50. | Yes |  | W16.2 | No | Diving or jumping into water |
| TC51. | Yes |  | W16.21 | No | Diving or jumping into water |
| TC52. | Yes |  | W16.22 | No | Diving or jumping into water |
| TC53. | No | On moving surface | W16.3 | No | Diving or jumping into water |
| TC5z. | Yes |  | W16.31 | No | Diving or jumping into water |
| TCy.. | Yes |  | W16.32 | No | Diving or jumping into water |
| TCy0. | No | Against other object | W16.33 | No | Diving or jumping into water |
| Tcyz. | No | other accidental fall | W16.4 | No | Diving or jumping into water |
| TCz.. | Yes |  | W16.41 | No | Diving or jumping into water |
| Xa1GP | Yes |  | W16.42 | No | Diving or jumping into water |
| XaISO | Yes |  | W16.5 | No | Diving or jumping into water |
| XaPC9 | No | No fear of falls | W16.51 | No | Diving or jumping into water |
| Ua1AN | Yes |  | W16.52 | No | Diving or jumping into water |
| Xaasz | Yes |  | W16.53 | No | Diving or jumping into water |
| Y7F24 | No | None coded | W16.6 | No | Diving or jumping into water |
|  |  |  | W16.61 | No | Diving or jumping into water |
|  |  |  | W16.62 | No | Diving or jumping into water |
|  |  |  | W16.7 | No | Diving or jumping into water |
|  |  |  | W16.71 | No | Diving or jumping into water |
|  |  |  | W16.72 | No | Diving or jumping into water |
|  |  |  | W16.8 | No | Diving or jumping into water |
|  |  |  | W16.81 | No | Diving or jumping into water |
|  |  |  | W16.82 | No | Diving or jumping into water |
|  |  |  | W16.83 | No | Diving or jumping into water |
|  |  |  | W16.9 | No | Diving or jumping into water |
|  |  |  | W16.91 | No | Diving or jumping into water |
|  |  |  | W16.92 | No | Diving or jumping into water |
|  |  |  | W17. | No | From one level to another |
|  |  |  | W17.1 | No | From one level to another |
|  |  |  | W17.2 | No | From one level to another |
|  |  |  | W17.3 | No | From one level to another |
|  |  |  | W17.4 | No | From one level to another |
|  |  |  | W17.8 | No | From one level to another |
|  |  |  | W17.81 | No | From one level to another |
|  |  |  | W17.82 | No | From one level to another |
|  |  |  | W17.89 | No | From one level to another |
|  |  |  | W18. | Yes |  |
|  |  |  | W18.0 | Yes |  |
|  |  |  | W18.00 | Yes |  |
|  |  |  | W18.01 | Yes |  |
|  |  |  | W18.02 | Yes |  |
|  |  |  | W18.09 | Yes |  |
|  |  |  | W18.1 | No | Involves Bath tub or shower |
|  |  |  | W18.11 | No | Involves Bath tub or shower |
|  |  |  | W18.12 | No | Involves Bath tub or shower |
|  |  |  | W18.2 | No |  |
|  |  |  | W18.3 | Yes |  |
|  |  |  | W18.30 | Yes |  |
|  |  |  | W18.31 | No |  |
|  |  |  | W18.39 | Yes |  |
|  |  |  | W18.4 | No |  |
|  |  |  | W18.40 | No |  |
|  |  |  | W18.41 | No |  |
|  |  |  | W18.42 | No |  |
|  |  |  | W18.43 | No |  |
|  |  |  | W18.49 | No |  |
|  |  |  | W19. | No | non-specific fall |
|  |  |  | W19.1 | Yes |  |
|  |  |  | W19.2 | Yes |  |
|  |  |  | W19.3 | Yes |  |
|  |  |  | W19.4 | Yes |  |
|  |  |  | W19.5 | Yes |  |
|  |  |  | W19.6 | Yes |  |
|  |  |  | W19.7 | Yes |  |
|  |  |  | W19.8 | Yes |  |
|  |  |  | W19.9 | Yes |  |
|  |  |  | W09.00 | No | Involves playground equipment |
|  |  |  | R29.6 | Yes |  |

Supplementary Table 2: Observational analysis in GP and HES derived fallers

|  | **GP Derived** | | | | | | | | **HES Derived** | | | | | | | |  |
| --- | --- | --- | --- | --- | --- | --- | --- | --- | --- | --- | --- | --- | --- | --- | --- | --- | --- |
| **Exposure** | **Binary** | | **Occurrence** | | | **Occurrence (ZIP)** | | | **Binary** | | **Occurrence** | | | **Occurrence (ZIP)** | | |  |
| Falls | OR (95% CI) | p-value | Beta | SE | p-value | Beta | SE | p-value | OR (95% CI) | p-value | Beta | SE | p-value | Beta | SE | p-value |  |
| Mean BMI* | 1.206 (1.187-1.225) | <2e-16 | 0.044 | 0.007 | 3.18E-09 | 0.130 | 0.014 | <2e-16 | 1.144 (1.130-1.158) | <2e-16 | 0.111 | 0.015 | 9.59E-14 | 0.098 | 0.006 | <2e-16 |  |
| Hand Grip Max* | 0.698 (0.672-0.725) | <2e-16 | -0.076 | 0.011 | 1.84E-12 | -0.211 | 0.020 | <2e-16 | 0.672 (0.654-0.691) | <2e-16 | -0.306 | 0.021 | <2e-16 | -0.244 | 0.007 | <2e-16 |  |
| Times measuring >100mg Physical Activity* | 0.887 (0.836-0.939) | 4.63E-06 | -0.048 | 0.015 | 0.001 | -0.181 | 0.044 | 4.51E-05 | 0.698 (0.658-0.737) | <2e-16 | -0.196 | 0.028 | 3.40E-12 | -0.269 | 0.020 | < 2e-16 |  |
| Times measuring >40mg Physical Activity* | 0.924 (0.873-0.975) | 0.002 | -0.044 | 0.015 | 0.003 | -0.166 | 0.045 | 1.96E-04 | 0.716 (0.677-0.755) | <2e-16 | -0.172 | 0.028 | 1.02E-09 | -0.235 | 0.020 | 1.96E-04 |  |
| International Physical Activity Questionnaire (IPAQ)* | 0.956 (0.936-0.976) | 1.37E-05 | -0.037 | 0.008 | 1.96E-06 | -0.100 | 0.016 | 1.98E-10 | 0.904 (0.890-0.919) | <2e-17 | -0.121 | 0.016 | 6.82E-14 | -0.105 | 0.006 | <2e-16 |  |
| Depressed Ever | 1.374 (1.290-1.459) | 1.75E-13 | 0.054 | 0.028 | 0.06 | 0.219 | 0.084 | 0.009 | 1.347 (1.280-1.415) | <2e-16 | 0.115 | 0.050 | 0.0199 | 0.204 | 0.044 | 3.70E-06 |  |
| CIDI Major Depressive Disorder Severity (CIDI MDD) | 1.050 (1.036-1.063) | 4.60E-13 | 0.010 | 0.004 | 1.55E-02 | 0.043 | 0.013 | 7.78E-04 | 1.052 (1.042-1.062) | <2e-16 | 0.034 | 0.007 | 4.45E-06 | 0.043 | 0.007 | <2e-16 |  |
| Public Health Questionaire Severity (PHQ9) | 1.053 (1.044-1.062) | <2e-16 | 0.007 | 0.003 | 0.013 | 0.029 | 0.009 | 0.001 | 1.073 (1.067-1.080) | <2e-16 | 0.040 | 0.005 | 3.31E-16 | 0.055 | 0.003 | <2e-16 |  |
| Generalised Anxiety Disorder (GAD7) | 1.040 (1.029-1.050) | 3.74E-14 | 0.006 | 0.003 | 0.10 | 0.021 | 0.010 | 0.045 | 1.053 (1.046-1.061) | <2e-16 | 0.031 | 0.005 | 2.71E-08 | 0.045 | 0.004 | 0.045 |  |
| GAD Ever | 1.654 (1.500-1.809) | 1.62E-10 | 0.102 | 0.050 | 0.040 | 0.410 | 0.143 | 0.004 | 1.704 (1.581-1.828) | <2e-16 | 0.244 | 0.087 | 0.005 | 0.440 | 0.075 | 4.03E-09 |  |
| Number of years in education | 0.974 (0.971-0.978) | <2e-16 | -0.011 | 0.002 | 1.77E-13 | -0.031 | 0.003 | <2e-16 | 0.969 (0.967-0.972) | <2e-16 | -0.020 | 0.003 | 3.48E-11 | -0.017 | 0.001 | <2e-16 |  |
| Units of alcohol consumption per week* | 0.952 (0.917-0.986) | 0.005 | -0.029 | 0.011 | 0.007 | -0.112 | 0.032 | 4.45E-04 | 0.987 (0.961-1.012) | 0.30 | -0.008 | 0.024 | 0.75 | -0.005 | 0.012 | 0.67 |  |
| Smoker status | 1.175 (1.146-1.203) | <2e-16 | 0.002 | 0.012 | 0.84 | 0.006 | 0.024 | 0.79 | 1.307 (1.287-1.328) | <2e-16 | 0.108 | 0.024 | 4.75E-06 | 0.092 | 0.009 | <2e-16 |  |
| Cigarettes per day* | 1.126 (1.105-1.147) | <2e-16 | 0.013 | 0.009 | 0.14 | 0.037 | 0.016 | 0.021 | 1.177 (1.162-1.191) | <2e-16 | 0.070 | 0.017 | 4.03E-05 | 0.058 | 0.006 | <2e-16 |  |
| *represents per change in standard deviation. All regressions using the binary falls metric as outcome uses a logistic model. All regressions using occurrence without controls metric uses a linear model. All regressions using the occurrence with controls uses a zero inflated poisson regression model. All models were adjusted for age and sex. | | | | | | | | | | | | | | | | |  |
|  |  |  |  |  |  |  |  |  |  |  |  |  |  |  |  |  |  |

| **Supplementary Table 3: Genetic Correlations of HES and GP derived falls against variables of interest.** | | | | | | |
| --- | --- | --- | --- | --- | --- | --- |
| **Genetic Correlations** | **GP Derived** | | | **HES Derived** | | |
| **Trait** | **rg*** | **SE** | **p-value** | **rg*** | **SE** | **p-value** |
| BMI | 0.3763 | 0.072 | 1.73E-07 | 0.3388 | 0.0497 | 9.12E-12 |
| Hand Grip Max | -0.2046 | 0.0642 | 0.001 | -0.2468 | 0.0449 | 3.77E-08 |
| Physucal Activity | -0.0864 | 0.0779 | 0.27 | -0.1727 | 0.0668 | 0.010 |
| Depression | 0.3763 | 0.072 | 1.73E-03 | 0.3606 | 0.0527 | 8.05E-12 |
| Anxiety | 0.589 | 0.273 | 0.031 | 0.3498 | 0.1768 | 0.048 |
| Neuroticism | 0.2899 | 0.0714 | 4.90E-05 | 0.2419 | 0.0504 | 1.62E-06 |
| Education years | -0.3108 | 0.0727 | 1.91E-05 | -0.2032 | 0.0455 | 7.85E-06 |
| Alzheimer’s | -0.1177 | 0.0932 | 0.21 | -0.0219 | 0.0736 | 0.77 |
| TDI | 0.3419 | 0.088 | 1.00E-04 | 0.3852 | 0.0716 | 7.39E-08 |
| Alcohol Consumption | 0.1157 | 0.1934 | 0.55 | 0.1928 | 0.0853 | 0.024 |
| Cigarettes per day | 0.3462 | 0.0677 | 3.11E-07 | 0.4945 | 0.0624 | 2.26E-15 |
| *rg is the proportion of the variance that two traits share due to genetic variants | | | | | | |

| **Supplementary Table 4: Top hits from Trajanoska et al (2020) in Falls GWAS** | | | | | | | |  |
| --- | --- | --- | --- | --- | --- | --- | --- | --- |
|  |  |  | **In the present GWASs** | | | | **In Trajanoska et al 2020** |  |
| **Metric** | **Source** | **SNP** | **MAF** | **Risk Increasing Allele (A1)** | **Beta** | **SE** | **P-Value** | **Risk Raising Allele** |
| Binary | Combined | rs2709062 | 0.501142 | G | -0.012 | 0.012 | 0.25 | A |
| Binary | HES | rs2709062 | 0.500822 | A | 0.003 | 0.010 | 0.80 | A |
| Binary | GP | rs2709062 | 0.500966 | G | -0.017 | 0.014 | 0.22 | A |
| Binary | Combined | rs2111530 | 0.606259 | G | -0.009 | 0.011 | 0.43 | G |
| Binary | HES | rs2111530 | 0.605839 | G | -0.017 | 0.010 | 0.08 | G |
| Binary | GP | rs2111530 | 0.606014 | G | -0.013 | 0.014 | 0.35 | G |
| SNPs are the two hits from the previous Falls GWAS based on self reported falls. These SNPs were identified in our GWAS and then the Betas, SE and P-Values were reported. | | | | | | | |  |

| **Supplementary Table 5: Sensitivity analysis stratifying the association of the top GWAS hit by age** | | | | | | |
| --- | --- | --- | --- | --- | --- | --- |
| Age | Cases | Controls | Risk of Falling per C Allele (OR) | LCI | UCL | P-Value |
| ≤65 | 13704 | 149650 | 1.055083594 | 1.018765 | 1.091402 | 3.82E-03 |
| >65 | 7085 | 31008 | 1.146518547 | 1.091874 | 1.201163 | 9.39E-07 |

| Supplementary Table 6: MR Egger P Values for all 2 Sample analyses | |
| --- | --- |
| **Exposure** | **MR Egger P Value** |
| BMI | 0.61 |
| Favourable Adiposity | 0.8 |
| Unfavourable Adiposity | 0.42 |
| Depressed Ever | 0.17 |
| Education years | 0.33 |
| Alcohol consumption | 0.21 |
|  |  |

| **Supplementary Table 7: MR lap results for HES and GP falls against phenotypes of interest.** | | | | | |
| --- | --- | --- | --- | --- | --- |
| **Exposure** | **HES/GP** | **Outcome** | **Primary MR Method** | **OR (95%CI)** | **P Value** |
| BMI | HES | Binary Falls | 2 -Sample | 1.329 (1.207-1.452) | 1.16E-05 |
| BMI | GP | Binary Falls | 2 -Sample | 1.235 (1.135-1.334) | 5.68E-05 |
| Favourable Adiposity | HES | Binary Falls | 2 -Sample | 1.324 (1.032-1.615) | 0.09 |
| Favourable Adiposity | GP | Binary Falls | 2 -Sample | 1.271 (0.899-1.644) | 0.24 |
| Unfavourable Adiposity | HES | Binary Falls | 2 -Sample | 1.489 (1.267-1.712) | 0.001 |
| Unfavourable Adiposity | GP | Binary Falls | 2 -Sample | 1.457 (1.216-1.699) | 0.004 |
| Hand Grip Strength Max | HES | Binary Falls | MRlap | 0.957 (0.943-0.971) | 1.17E-09 |
| Hand Grip Strength Max | GP | Binary Falls | MRlap | 0.955 (0.936-0.974) | 2.27E-06 |
| Physical Activity | HES | Binary Falls | MRlap | 0.973 (0.949-0.997) | 0.024 |
| Physical Activity | GP | Binary Falls | MRlap | 0.993 (0.959-1.027) | 0.67 |
| Sedentary Time | HES | Binary Falls | MRlap | 1.005 (0.973-1.038) | 0.47 |
| Sedentary Time | GP | Binary Falls | MRlap | 1.061 (1.014-1.109) | 0.015 |
| Depressed Ever | HES | Binary Falls | 2 -Sample | 1.141 (0.927-1.354) | 0.24 |
| Depressed Ever | GP | Binary Falls | 2 -Sample | 1.254 (0.952-1.556) | 0.162 |
| Neuroticism | HES | Binary Falls | MRlap | 1.050 (1.022-1.077) | 5.28E-04 |
| Neuroticism | GP | Binary Falls | MRlap | 1.080 (1.044-1.116) | 2.39E-05 |
| Generalised Anxiety | HES | Binary Falls | MRlap | 1.023 (0.911-1.135) | 0.69 |
| Generalised Anxiety | GP | Binary Falls | MRlap | 1.093 (0.922-1.263) | 0.31 |
| PGC Major Depressive Disorder | HES | Binary Falls | MRlap | 1.083 (1.034-1.131) | 2.92E-05 |
| PGC Major Depressive Disorder | GP | Binary Falls | MRlap | 1.135 (1.073-1.197) | 2.92E-05 |
| education years | HES | Binary Falls | MRlap | 0.924 (0.905-0.944) | 6.47E-06 |
| education years | GP | Binary Falls | MRlap | 0.924 (0.896-0.953) | 6.57E-04 |
| Mean units of alcohol consumption per week | HES | Binary Falls | 2 -Sample | 2.424 (1.855-2.992) | 0.028 |
| Mean units of alcohol consumption per week | GP | Binary Falls | 2 -Sample | 0.815 (0.202-1.428) | 0.54 |

| **Supplementary table 8 One Sample MR results** | | | | | | |
| --- | --- | --- | --- | --- | --- | --- |
| **Exposure** | **Outcome** | **RR (95% CI)** | **Sample Size** | **SNPs** | **F-statistic** | **R^2^** |
| BMI | Binary Falls | 1.223 (1.147- 1.343) | 430,944 | 73 | 102.6774 | 0.0169 |
| Hand Grip Strength | Binary Falls | 0.740 (0.670- 0.819) | 429,014 | 192 | 20.5502 | 0.0134 |
| CIDI MDD | Binary Falls | 1.330 (1.153- 1.346) | 140,873 | Levey GRS | 756.5869 | 0.0053 |
| PHQ9 | Binary Falls | 1.342 (1.178- 1.528) | 140,873 | Levey GRS | 416.3891 | 0.0029 |
| Alcohol | Binary Falls | 1.786 (1.584- 2.014) | 328,194 | 19 | 72.3847 | 0.0042 |
| Summary of Genetic Variants used in one-sample MR analyses. Causal effects as estimated by a Poisson model, results are converted in rate ratio (95% CI), interpreted as how many times the rate of falls changes in response to a unit change in the exposure. Alcohol, BMI, grip strength are standardised. | | | | | | |

| Supplementary Figure 1: Histogram of Continuous falls distribution in combined, HES and GP falls measures |
| --- |
| 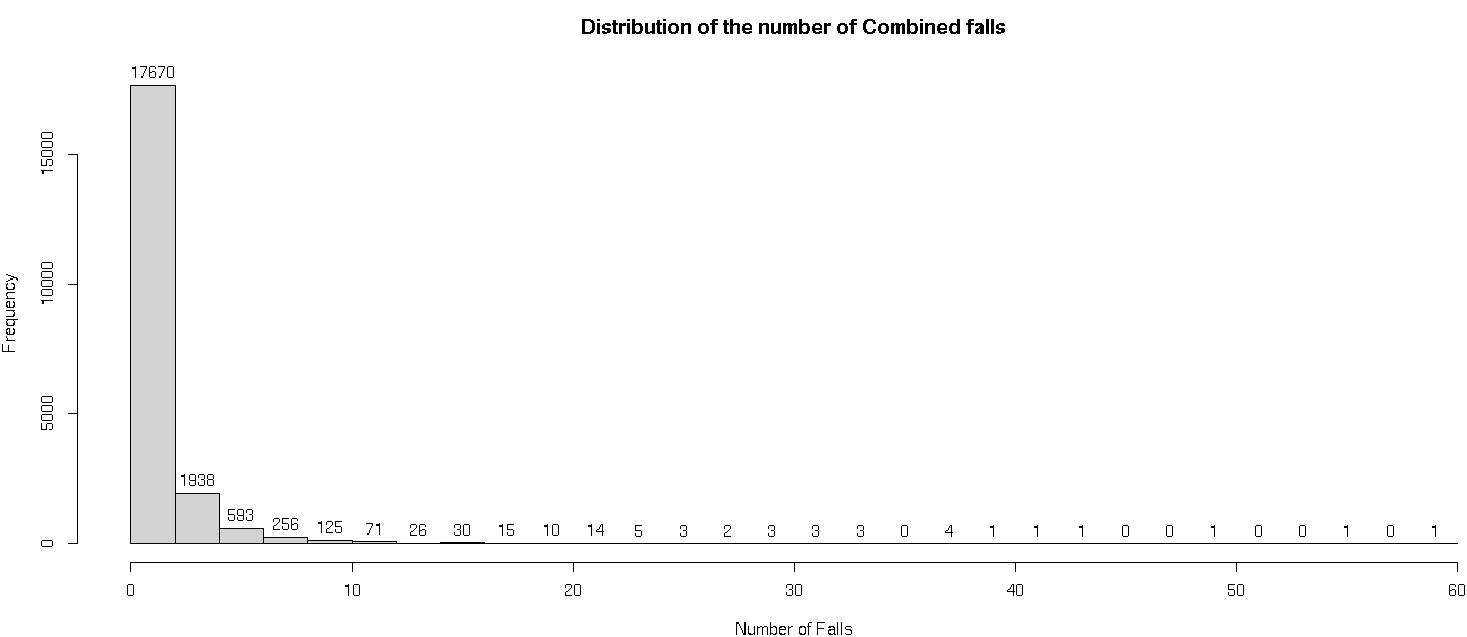 |
| 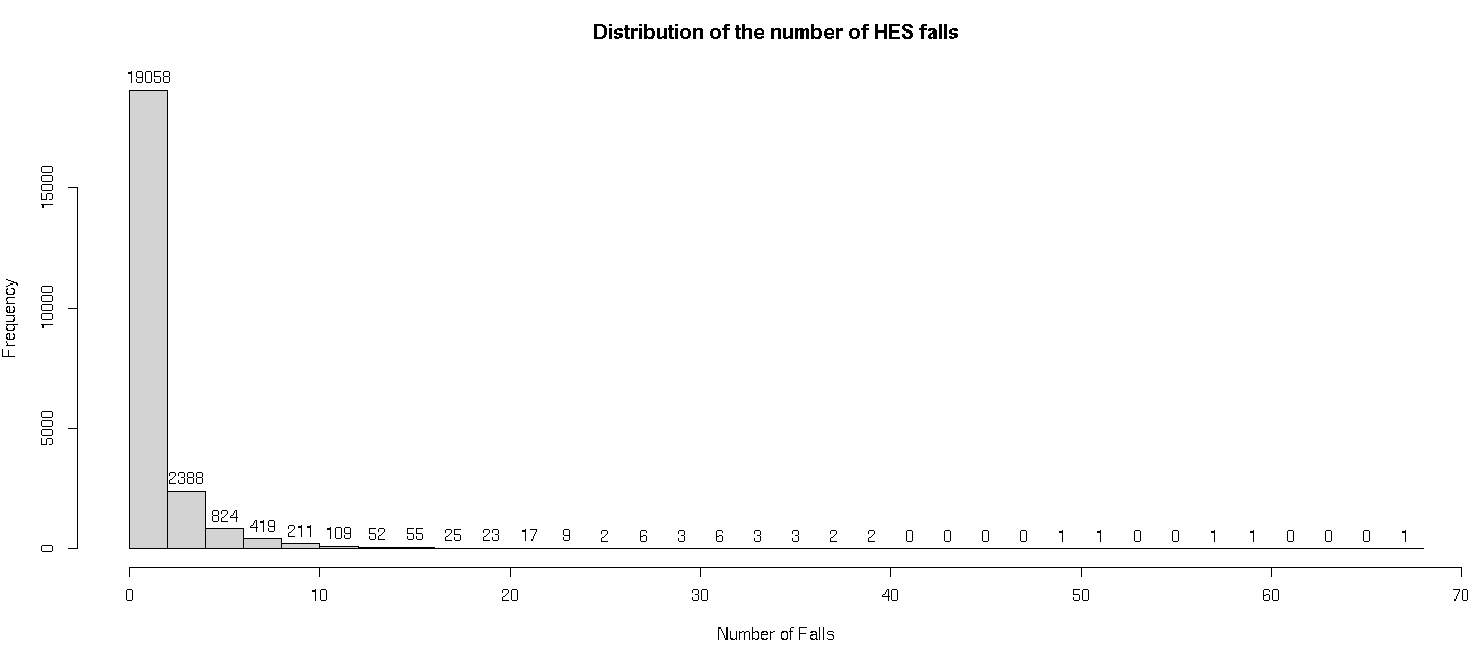 |
| 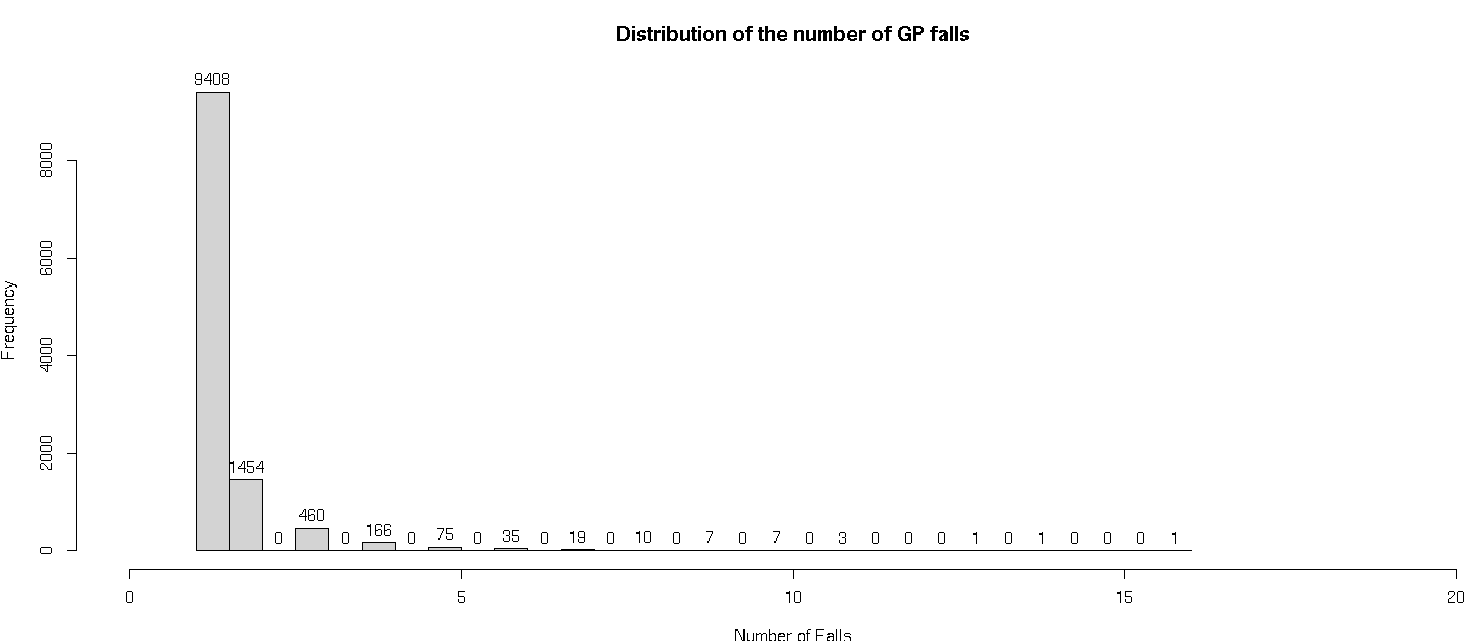 |

| Supplementary Figure 2: Manhattan plots of Continuous Combined, HES and GP and Binary HES and GP GWASs. | | |
| --- | --- | --- |
| 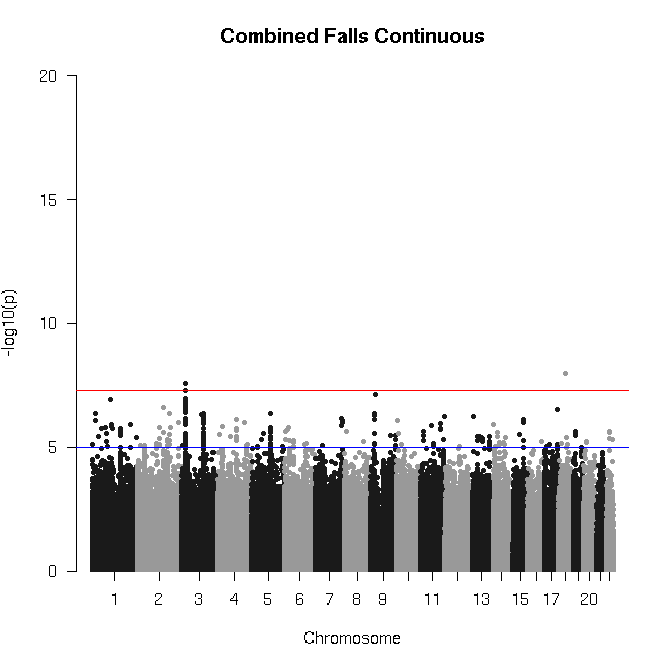 | 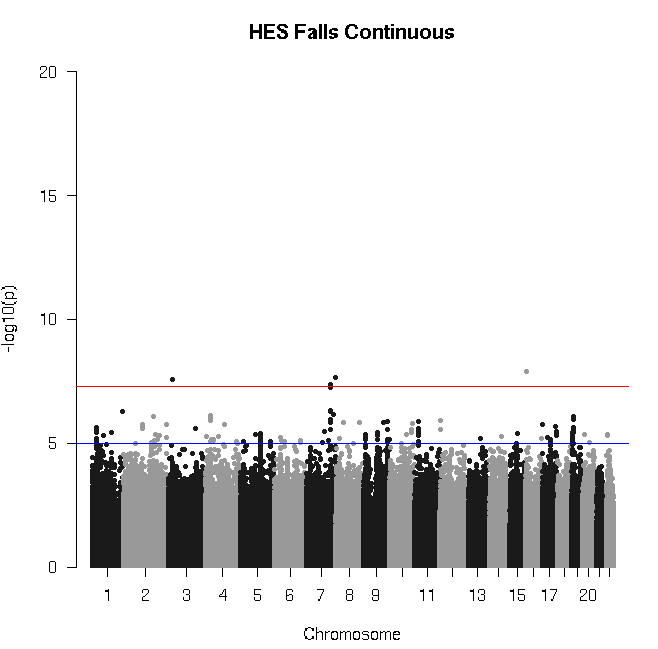 | 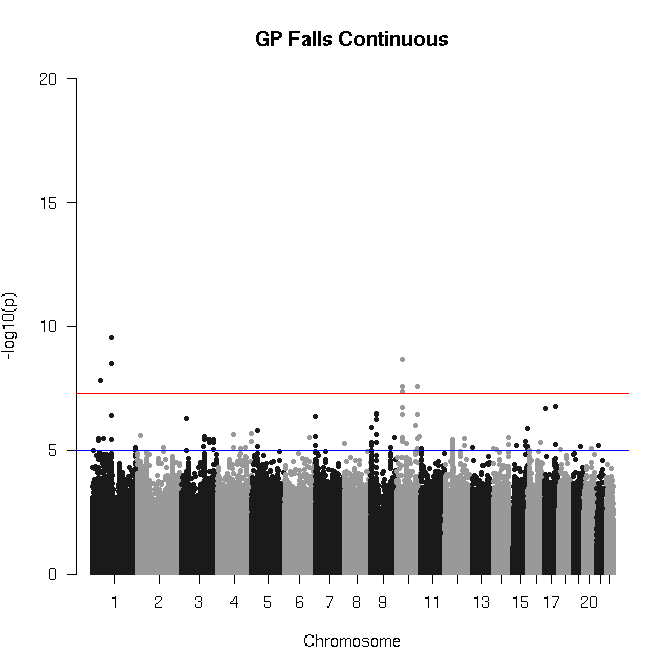 |
| 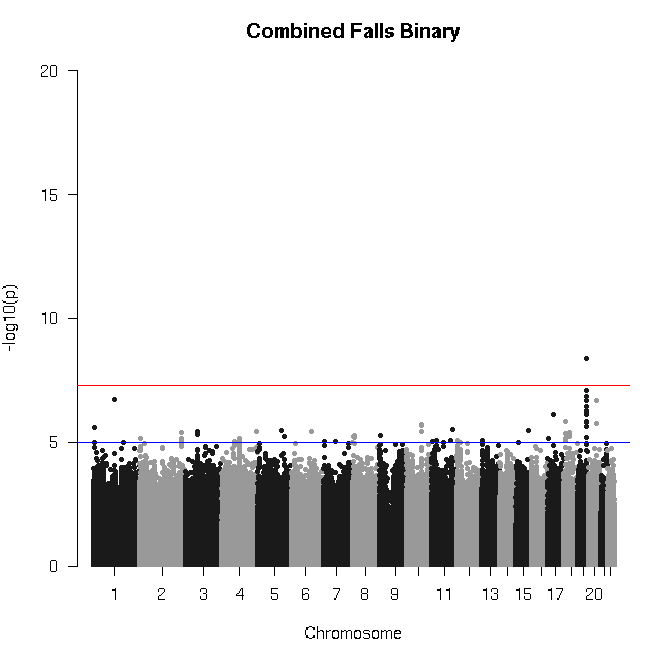 | 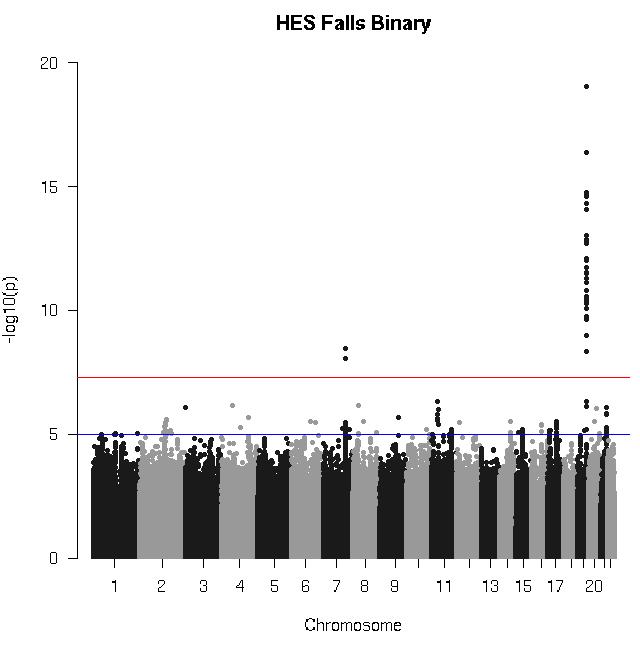 | 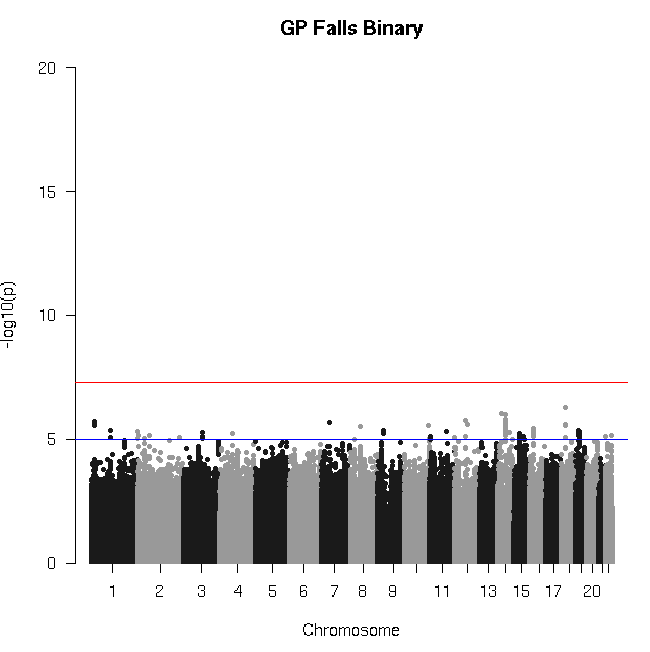 |

| 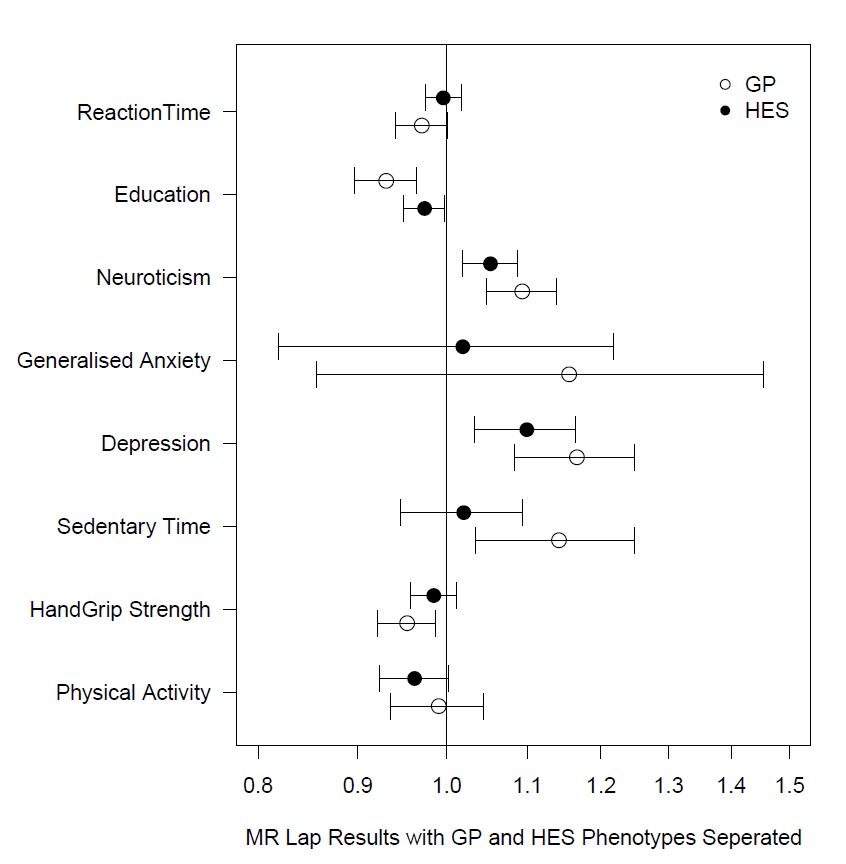 |
| --- |
| Supplementary Figure 3: MR Lap results when conducting analysis on the HES derived and GP derived falls separately. Binary falls is treated as the exposure. |
|  |
|  |
